# Diagnostic Value of the Delta Neutrophil Index and Neutrophil-to-Lymphocyte Ratio for Preoperative Differentiation of Malignant and Benign Primary Brain Tumors: A Retrospective Cohort Study

**DOI:** 10.3390/brainsci16020169

**Published:** 2026-01-30

**Authors:** Emrullah Cem Kesilmez, Muharrem Furkan Yüzbaşı, Muhammed Kırkgeçit, Hasan Türkoğlu, Kasım Zafer Yüksel

**Affiliations:** 1 Neurosurgery Clinic, Kahramanmaraş Sütçü İmam University, Kahramanmaraş 46050, Türkiye; 2 Neurosurgery Clinic, MegaPoint Hospital, Kahramanmaraş 46100, Türkiyehas.1453@hotmail.com (H.T.); 3 Neurosurgery Clinic, Gaziantep City Hospital, Gaziantep 27470, Türkiye

**Keywords:** biomarkers, blood cell count, brain neoplasms, inflammation, preoperative period, diagnostic tests

## Abstract

**Aim:** This study aimed to evaluate the diagnostic performance of the Delta Neutrophil Index (DNI) and Neutrophil-to-Lymphocyte Ratio (NLR) in distinguishing malignant from benign primary brain tumors during the preoperative period. **Methods:** This retrospective cohort study was conducted at a tertiary university hospital. A total of 140 participants were included 60 patients with malignant glial tumors, 50 patients with benign brain tumors, and 30 healthy controls without inflammatory, infectious, or hematologic disease. Preoperative complete blood count results obtained within seven days before surgery were analyzed. **Results:** Patients with malignant tumors were significantly older than those in the benign and control groups (*p* < 0.001). DNI, NLR, PLR, MLR, and SII values were all significantly elevated in the malignant group (*p* < 0.001, for all comparisons). ROC analysis revealed high diagnostic accuracy for DNI (AUC = 0.847) and NLR (AUC = 0.850), with optimal cut-off values of 3.50 and 3.95, respectively. In multivariable logistic regression adjusted for age, DNI > 3.5 (OR = 20.67; 95% CI: 3.35–127.64; *p* = 0.001), NLR > 3.95 (OR = 21.17; 95% CI: 3.28–136.50; *p* = 0.001), and CRP (OR = 1.52; 95% CI: 1.20–1.93; *p* = 0.001) remained independent predictors of malignancy. The combined model including DNI and NLR achieved the highest diagnostic accuracy (AUC = 0.937; age-adjusted AUC = 0.943), with a sensitivity of 88.3% and a specificity of 86.0% after age adjustment. **Conclusions:** Both DNI and NLR demonstrated significant value in differentiating malignant from benign primary brain tumors prior to surgery, with DNI emerging as the most powerful independent predictor. The combined use of DNI and NLR substantially improved diagnostic accuracy, suggesting that simple hematologic indices may serve as practical, noninvasive adjunctive tools in the preoperative assessment of brain tumor malignancy. These markers may assist in surgical prioritization, patient counseling, and clinical decision-making, particularly in resource-limited settings.

## 1. Introduction

Primary brain tumors constitute a heterogeneous group of disorders associated with substantial morbidity and mortality and directly compromise neurological function. These tumors may present with a wide spectrum of similar clinical and radiological features and distinguishing malignant from benign lesions in the preoperative setting may be challenging in some cases [1]. Early diagnosis and appropriate surgical planning are crucial for optimizing survival, postoperative neurological outcomes, and guiding adjuvant therapeutic strategies [2,3]. Preoperative prediction of malignancy may influence several aspects of clinical management, including the urgency of surgical intervention, the extent of planned resection, patient counseling regarding prognosis, and the coordination of multidisciplinary oncologic resources. In settings where advanced imaging modalities such as perfusion MRI or MR spectroscopy are unavailable, simple biomarkers that indicate the likelihood of malignancy could facilitate more informed clinical decision-making and appropriate referral pathways. Although conventional imaging modalities provide valuable information in most patients, their diagnostic accuracy is sometimes limited in differentiating malignant tumors from benign lesions due to reactive changes such as inflammation and edema [1,4]. Therefore, there is a need for easily accessible, cost-effective, and practical biomarkers capable of predicting the likelihood of malignancy in the preoperative period. Such biomarkers could prove particularly valuable in triaging patients for expedited surgical scheduling, guiding discussions about intraoperative frozen section necessity, and informing preoperative conversations with patients and families about the probable nature of their disease.

Systemic inflammation has been shown to play a key role in tumor development, and the peripheral immune response is known to reflect tumor biology through alterations in circulating blood parameters [5,6]. Hematological inflammation indices derived from complete blood count measurements have therefore become widely investigated in clinical practice. The neutrophil-to-lymphocyte ratio (NLR), platelet-to-lymphocyte ratio (PLR), monocyte-to-lymphocyte ratio (MLR), and systemic immune-inflammation index (SII) are among the most frequently studied markers associated with prognosis in solid tumors [5,7,8]. In contrast, the Delta Neutrophil Index (DNI), which indicates the proportion of immature granulocytes in circulation, has primarily been investigated in sepsis, gastrointestinal disorders, trauma, and patients in emergency or intensive care settings, and data regarding its potential role in the preoperative evaluation of tumors remain limited [9,10,11,12,13]. Its association with differentiation and prognosis has been reported in several malignancies, including renal cell carcinoma, thyroid cancer, and breast cancer [14,15,16]. Although malignant brain tumors are known to elicit a neutrophil-predominant inflammatory response, the extent to which this response is reflected in peripheral inflammatory indices remains uncertain [17]. For this reason, evaluating the diagnostic value of simple hematological parameters such as DNI and NLR represents a clinically and scientifically relevant area of interest.

This study aims to evaluate the diagnostic performance of selected blood count parameters and their derivatives, including NLR, PLR, MLR, and SII, in distinguishing malignant from benign primary brain tumors in the preoperative period, and to determine whether these indices have the potential to independently predict malignancy.

## 2. Methods

### 2.1. Study Design and Ethical Approval

This retrospective cohort study included patients who underwent surgery for intracranial masses between January 2021 and August 2024. The study was conducted in the Department of Neurosurgery at Kahramanmaraş Sütçü İmam University Faculty of Medicine Hospital. The study protocol was approved by the Kahramanmaraş Sütçü İmam University Clinical Research Ethics Committee (Decision No: 2021/24/7, Date: 6 July 2021). Due to the retrospective study design, written informed consent was not obtained from the participants. The study was carried out in accordance with the principles of the Declaration of Helsinki.

### 2.2. Study Population and Grouping

The study included adult patients who underwent surgery for intracranial masses and had complete available preoperative laboratory data. A total of 110 patients were evaluated and classified into three groups based on their histopathological diagnosis. The malignant group consisted of 60 patients diagnosed with glioblastoma or anaplastic astrocytoma, and the benign group consisted of 50 patients diagnosed with meningioma, schwannoma, or pituitary adenoma. The control group included 30 healthy volunteers who presented to our hospital for routine health evaluation and had no systemic or chronic medical conditions. All diagnoses were confirmed by postoperative histopathological examination. Surgical records, pathology reports, and laboratory findings of all cases were reviewed in detail. Patients with recurrent or metastatic tumors and those with incomplete clinical or laboratory data were excluded from the study.

### 2.3. Inclusion and Exclusion Criteria

The inclusion criteria were being 18 years of age or older, undergoing elective surgery for an intracranial mass, having a complete blood count (CBC) obtained within seven days before the operation, and having a confirmed histopathological diagnosis. Patients with clinical signs of active infection or recent antibiotic use, those with chronic inflammatory, autoimmune, or hematologic diseases, those who had received a blood transfusion within the previous four weeks, patients with confirmed pregnancy, and individuals receiving high-dose corticosteroid therapy were excluded from the study. Medication history including corticosteroid, antibiotic, and nonsteroidal anti-inflammatory drug (NSAID) use was documented for all participants. Patients with recent antibiotic use within seven days before blood sampling were excluded. Corticosteroid and NSAID use were recorded and included as covariates in the statistical analyses to control for their potential effects on hematological parameters.

### 2.4. Data Collection

Demographic characteristics, clinical findings, tumor type, and histopathological classification of all patients were retrieved from the electronic medical record system. Radiological data were verified using preoperative magnetic resonance imaging reports, and the presence of primary intracranial lesions was confirmed in all cases. The control group consisted of individuals who underwent routine health examinations at the same institution, were screened for systemic disease through physical examination and laboratory assessments, and had CBC testing performed at the time of evaluation.

Laboratory data were obtained through the institutional biochemistry laboratory information system. Blood samples were drawn from the antecubital vein in all subjects during morning hours following an overnight fast, collected into ethylenediaminetetraacetic acid tubes, and analyzed within two hours.

### 2.5. Laboratory Analysis and Calculation of Hematological Indices

CBC analyses were performed in the central laboratory of the hospital using the Sysmex XN-1000 automated hematology analyzer (Sysmex Corporation, Kobe, Japan). The device was calibrated daily according to the manufacturer’s recommendations and used after completion of routine quality control procedures. White blood cell count, neutrophil count, lymphocyte count, monocyte count, platelet count, hemoglobin, hematocrit, and DNI values were recorded.

DNI is an automatically calculated parameter based on the difference between measurements obtained from the myeloperoxidase and nuclear lobularity channels and reflects the proportion of immature granulocytes in circulation.

Derived hematological indices including NLR, PLR, MLR, and SII (platelet × neutrophil/lymphocyte) were calculated using the measured parameters.

### 2.6. Statistical Analysis

All analyses were conducted using IBM SPSS version 27.0 (IBM Corp., Armonk, NY, USA). *p* < 0.05 values were accepted as statistically significant results. Normality assumption was evaluated using histograms and Q–Q plots. Descriptive statistics were presented using mean ± standard deviation for normally distributed continuous variables, median (25th percentile–75th percentile) for non-normally distributed continuous variables and frequency (percentage) for categorical variables. Between-groups analysis of continuous variables were performed using the one-way analysis of variance (ANOVA) or Kruskal–Wallis test depending on the normality of distribution. Between-groups analysis of categorical variables were performed using the chi-square test or Fisher–Freeman–Halton test. Pairwise comparisons were adjusted using the Bonferroni correction method. The performance of variables to discriminate malign and benign tumors were evaluated using the receiver operating characteristic (ROC) curve analysis. Optimal cut-off points were determined using Youden’s index. In addition, age-adjusted ROC curve analysis was performed using propensity scores. Multivariable logistic regression analysis was used to construct a model consisting of a combination of DNI and NLR to discriminate malign and benign tumors. In addition, logistic regression analyses were performed to determine significant factors that were independently associated with malignancy. Variables were analyzed using univariable logistic regression analysis, then statistically significant variables were included in multivariable logistic regression analysis, which was performed via the forward conditional selection method to avoid multicollinearity. In addition, the final multivariable model was adjusted by age since it is a potential confounding factor. Multicollinearity assessment was performed by calculating variance inflation factors.

## 3. Results

A total of 140 participants were included in the study, comprising 60 patients with malignant glial tumors, 50 patients with benign brain tumors, and 30 healthy controls. The median age differed significantly among the groups (*p* < 0.001). Patients in the malignant tumor group were older than those in the benign group, whereas no significant age difference was observed between the malignant and control groups. The sex distribution was comparable across all groups (*p* = 0.499).

Regarding medication use, preoperative corticosteroid therapy was documented in 16 patients (26.7%) in the malignant group, 9 patients (18.0%) in the benign group, and 11 individuals (36.7%) in the control group, with no significant difference among groups (*p* = 0.176). NSAID use was similarly distributed across the malignant (21.7%), benign (20.0%), and control (30.0%) groups (*p* = 0.563). None of the participants had received antibiotics within seven days prior to blood sampling.

Preoperative laboratory parameters showed distinct patterns between groups. Total white blood cell and neutrophil counts were significantly higher in the malignant group compared with both the benign and control groups (*p* < 0.001 for all). Conversely, lymphocyte counts were significantly lower in malignant cases (1.27 ± 0.50 × 10^9^/L) than in the benign (1.71 ± 0.49 × 10^9^/L) and control groups (1.97 ± 0.61 × 10^9^/L) (*p* < 0.001). Monocyte, platelet, hemoglobin, and hematocrit values did not differ significantly among the groups (all *p* > 0.05). C-reactive protein levels were significantly elevated in the malignant group (median 10.13 mg/L, IQR 7.32–13.38) compared to the benign group (median 4.56 mg/L, IQR 2.99–5.60) (*p* < 0.001). Notably, CRP levels in the control group (median 8.60 mg/L, IQR 3.53–10.39) were intermediate and significantly higher than those in the benign group. Serum albumin levels were highest in the benign group (3.99 ± 0.32 g/dL) and lowest in the control group (3.64 ± 0.30 g/dL), with the malignant group showing intermediate values (3.72 ± 0.33 g/dL) (*p* < 0.001).

DNI demonstrated a marked difference, showing the highest mean value in the malignant group (5.00 ± 1.99%), followed by the benign (2.51 ± 1.28%) and control (1.08 ± 0.53%) groups (*p* < 0.001). Similarly, inflammation-related ratios, including NLR, PLR, MLR, and SII, were significantly elevated in the malignant group compared to both benign and control subjects (all *p* < 0.001) (Table 1).

**Figure 1:**
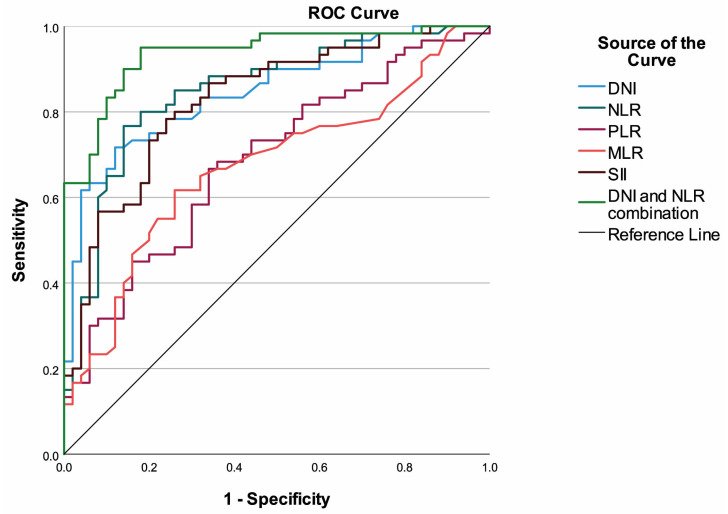
ROC curves of the markers to discriminate malign and benign tumors.

ROC curve analysis demonstrated that both DNI and NLR were strong discriminators between malignant and benign brain tumors. The optimal cut-off value for DNI was determined as 3.50, yielding a sensitivity of 71.7% and specificity of 88.0% with an AUC of 0.847 (95% CI, 0.775–0.919; *p* < 0.001). Similarly, an NLR threshold of 3.95 provided a sensitivity of 76.7% and specificity of 86.0%, corresponding to an AUC of 0.850 (95% CI, 0.776–0.923; *p* < 0.001). Both indices outperformed other hematologic markers in distinguishing malignant from benign lesions. Among the remaining parameters, SII also demonstrated a relatively high diagnostic performance with an AUC of 0.829 (95% CI, 0.752–0.905; *p* < 0.001), whereas PLR and MLR yielded lower discriminatory capacities (AUC = 0.688 and 0.674, respectively). The combination of DNI and NLR using a logistic regression model further improved predictive accuracy, achieving an AUC of 0.937 (95% CI, 0.893–0.981) with 85.0% sensitivity and 86.0% specificity. This composite model provided the highest overall accuracy (85.5%), outperforming each parameter used individually (Table 2, Figure 1).

**Figure 2:**
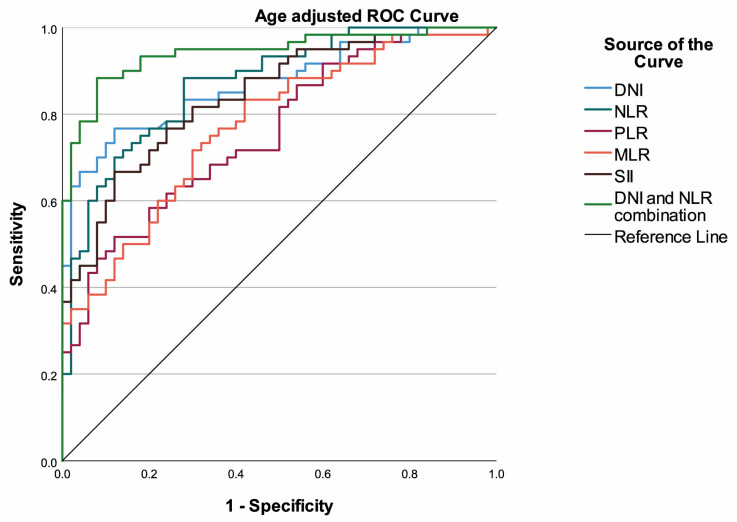
Age-adjusted ROC curves of the markers to discriminate malign and benign tumors.

To address the potential confounding effect of age on diagnostic performance, age-adjusted ROC curve analyses were performed using propensity scores. After adjustment for age, both DNI and NLR maintained excellent discriminative ability, with AUC values of 0.870 (95% CI: 0.804–0.935) for both markers. The combined DNI-NLR model showed marginally improved performance after age adjustment, achieving an AUC of 0.943 (95% CI: 0.900–0.986) with a sensitivity of 88.3% and a specificity of 86.0%. These findings confirm that the diagnostic utility of DNI and NLR is not solely attributable to age-related differences between groups (Table 3, Figure 2).

According to multivariable logistic regression analysis results, high white blood cell count (OR: 1.878, 95% CI: 1.160–3.041, *p* = 0.010), high DNI (OR: 20.667, 95% CI: 3.346–127.642, *p* = 0.001), high NLR (OR: 21.165, 95% CI: 3.282–136.501, *p* = 0.001) and high CRP (OR: 1.522, 95% CI: 1.200–1.931, *p* = 0.001) were independently associated with malign tumors after being adjusted by age (*p* = 0.063). Other variables included in the multivariable analysis, neutrophil count (*p* = 0.108), lymphocyte count (*p* = 0.693), PLR (*p* = 0.662), MLR (*p* = 0.369), SII (*p* = 0.978) and albumin (*p* = 0.701), were found to be non-significant. The highest variance inflation factor was 1.371 (for NLR), which means there was no multicollinearity in the final model (Table 4).

## 4. Discussion

In this study, the potential role of hematological inflammation markers in distinguishing malignant from benign primary brain tumors during the preoperative period was investigated. The results demonstrated significant associations between malignancy and age, DNI, and NLR values. The findings indicate that age contributes to an increased risk of malignancy despite its limited predictive strength, DNI emerges as the strongest independent predictor, and NLR provides a more consistent discriminatory value compared with other inflammatory indices.

Although the presence of malignant brain tumors was found to be significantly associated with age in this study, its contribution appeared to be more limited compared with the hematological markers in multivariable analyses. The observation that patients in the malignant group were significantly older than those in the benign group is consistent with the literature reporting a higher incidence of high-grade glial tumors in advanced age [18]. This relationship has been attributed to mechanisms such as age-related immune dysfunction, persistent low-grade inflammatory responses, and the accumulation of genetic mutations over time [19,20]. However, some studies have also reported that gliomas may occur in younger individuals [21]. These discrepancies may be related to characteristics of the study population, histopathological subtypes of tumors, and molecular or genetic variations. Increasing exposure to carcinogens and risk factors with advancing age may also contribute to this association [19]. Given that systemic inflammatory markers, including DNI and NLR, have been reported to increase with advancing age in the general population, we performed age-adjusted ROC analyses to evaluate whether the observed diagnostic performance was confounded by age differences between groups. Importantly, both DNI and NLR retained strong discriminative ability after age adjustment, with AUC values of 0.870 for each marker. Furthermore, age did not achieve statistical significance in the final multivariable model (*p* = 0.063) when included alongside DNI, NLR, WBC, and CRP, suggesting that these inflammatory indices provide diagnostic information independent of age. Nevertheless, the establishment of age-specific reference ranges for these markers may enhance their clinical applicability in neuro-oncology practice.

DNI is a parameter that reflects the proportion of immature granulocytes in circulation and is based on the quantitative detection of immature granulocytes released into peripheral blood during inflammatory states characterized by cytokine-mediated neutrophil proliferation [22]. An interesting finding in our study was that DNI and neutrophil counts were also elevated in the benign tumor group compared to healthy controls, although to a lesser extent than in malignant cases. Several mechanisms may explain this observation. First, even benign intracranial tumors can trigger local inflammatory responses through pressure on surrounding brain tissue and disruption of the blood–brain barrier, which may lead to mild systemic inflammation [23]. Second, peritumoral edema, which is commonly seen in benign tumors such as meningiomas, is associated with the release of inflammatory cytokines, including interleukin-6 and tumor necrosis factor-alpha, which can stimulate the production of white blood cells in bone marrow [24]. Third, the stress of having a symptomatic brain lesion, regardless of tumor grade, may activate the body’s stress response system, causing neutrophils to be released into the bloodstream [25]. These findings suggest that the presence of any intracranial mass, whether malignant or benign, may cause detectable changes in blood inflammatory markers, with the degree of change reflecting tumor aggressiveness.

The observation that benign tumors—predominantly meningiomas in our cohort—exhibited elevated inflammatory markers compared to healthy controls warrants further consideration, particularly given that such elevations may not align with routine clinical experience. Several factors may explain this apparent discrepancy. First, our study population consisted exclusively of patients undergoing elective craniotomy, representing a selected subset with tumors sufficiently symptomatic or large enough to warrant surgical intervention. Indeed, 82% of patients in the benign group presented with at least one neurological symptom, indicating clinically significant lesions rather than small, incidentally discovered tumors. This selection toward symptomatic surgical candidates likely enriched our cohort for tumors with greater mass effect, more extensive peritumoral edema, or proximity to eloquent brain regions—features that have been associated with heightened local and systemic inflammatory responses. Second, even histologically benign meningiomas demonstrate considerable biological heterogeneity, with some tumors exhibiting aggressive growth patterns, brain invasion, or secretory phenotypes associated with substantial peritumoral edema and cytokine release. Prior studies have demonstrated that meningiomas with prominent peritumoral edema show increased expression of vascular endothelial growth factor and elevated serum interleukin-6 levels, both of which can stimulate hepatic acute-phase responses and bone marrow granulopoiesis. Third, the preoperative setting itself—characterized by psychological stress, altered sleep patterns, and in some cases, reduced oral intake—may contribute to mild neutrophilia through activation of the hypothalamic–pituitary–adrenal axis. These factors, acting in concert, may explain why our surgical cohort demonstrated detectable inflammatory marker elevations that would not be expected in the general population of patients with small, asymptomatic, and conservatively managed meningiomas.

DNI’s automated calculation by hematology analyzers reduces observer-dependent variability and enhances its applicability in clinical practice. In this study, among all evaluated hematological parameters, DNI demonstrated the highest diagnostic accuracy in predicting malignant brain tumors and was also identified as the strongest independent predictor of malignancy in multivariable analyses. This finding suggests that DNI may serve as an easily accessible, inexpensive, and effective biomarker that could contribute meaningfully to preoperative clinical decision-making. Previous reports have indicated that DNI has diagnostic and prognostic utility in infection-related inflammatory conditions, particularly in sepsis and gastrointestinal disorders. Furthermore, its accuracy has been reported to improve when evaluated in conjunction with other inflammatory indices rather than as a single biomarker [9,22,26]. Similarly, our results showed that DNI independently predicted the presence of malignancy and that its combined use with NLR significantly enhanced diagnostic performance.

Studies conducted in solid organ tumors have also demonstrated an association between DNI and both tumor aggressiveness and metastatic potential. In patients with breast cancer, DNI has been shown to predict axillary metastasis preoperatively with high accuracy [15]. Similarly, in renal cell carcinoma, higher DNI values have been correlated with advanced tumor stage and identified as an independent predictor when evaluated alongside NLR [16]. Additionally, DNI has been reported to exhibit strong diagnostic performance in distinguishing thyroid malignancies from benign nodules [14]. The identification of DNI as the strongest independent predictor in malignant primary brain tumors in our study further supports its consistent diagnostic value across different tumor types. Reports indicating higher DNI levels in advanced-stage and high-grade tumors suggest that this parameter may reflect key biological features associated with aggressive tumor phenotypes. Central nervous system malignancies are characterized by persistent activation of inflammatory signaling pathways such as STAT3 and NF-κB. Tumor-promoting inflammation is sustained through the contribution of microglia, macrophages, and reactive astrocytes [27]. This chronic inflammatory microenvironment may facilitate immune evasion mechanisms and lead to a neutrophil-dominant profile in peripheral blood parameters. The elevated DNI and NLR levels observed in malignant cases in our study may represent a systemic reflection of this underlying pathophysiology.

In our study, NLR maintained its significance as an independent predictor of malignancy along with DNI. Previous studies have demonstrated that NLR correlates directly with glioma grade and provides high diagnostic accuracy in differentiating malignant tumors [21,28]. A comprehensive meta-analysis showed that elevated NLR levels in patients with glioblastoma were associated with poor overall survival and progression-free survival. PLR was also found to have prognostic relevance, whereas other indices, including SII and SIRI, did not demonstrate significant value [29]. Similarly, a systematic review on meningiomas reported that higher NLR levels were significantly associated with high-grade tumors and carried prognostic implications [30]. Comparable results have also been demonstrated in pediatric populations [31]. Another study found that elevated preoperative NLR levels correlated not only with tumor grade but also with anatomical localization, particularly in intra-axial tumors [32]. Research comparing different intracranial pathologies has also indicated that glial and metastatic solid tumors are associated with significant increases in neutrophil and platelet counts, accompanied by higher NLR and PLR levels. This phenomenon has been interpreted as a peripheral reflection of systemic inflammatory activation in solid brain tumors, and PLR has been reported to have diagnostic value in differentiating metastases from glioblastoma [33]. These observations suggest that NLR may reflect both the biological aggressiveness and localization of central nervous system tumors. However, there are studies reporting that NLR does not correlate with survival or disease stage in glioblastoma [17,34]. Taken together, the existing evidence indicates that NLR has diagnostic and prognostic potential as a practical biomarker in brain tumors, although its performance may vary depending on patient heterogeneity and methodological differences across studies. Clinical application of NLR should therefore be interpreted in conjunction with additional parameters and supported by large-scale prospective investigations. In our study, although PLR and MLR exhibited significance in univariable analysis, they did not remain independent predictors in multivariable evaluation. This is consistent with the heterogeneity and prognostic uncertainty reported in the literature regarding these markers [35,36]. These findings suggest that PLR and MLR may provide supportive information during diagnostic assessment but are insufficient as sole determinants in clinical decision-making.

The inclusion of CRP and albumin in our analysis provided additional insights into the inflammatory and nutritional milieu associated with brain tumor malignancy. CRP, an acute-phase reactant synthesized in response to inflammatory cytokines, was significantly elevated in malignant tumors and emerged as an independent predictor of malignancy alongside DNI and NLR. This finding aligns with previous reports demonstrating that CRP reflects tumor-associated systemic inflammation and carries prognostic significance in various malignancies [37]. Interestingly, serum albumin, often considered a marker of nutritional status and chronic inflammation, did not independently predict malignancy in our multivariable model despite significant univariable differences. This suggests that the inflammatory response, rather than nutritional depletion, is the predominant driver of hematological alterations in malignant brain tumors.

The clinical implications of these findings merit consideration. First, in healthcare settings where advanced neuroimaging techniques such as perfusion-weighted MRI, MR spectroscopy, or positron emission tomography are not readily available, DNI and NLR could serve as accessible adjunctive tools to support clinical suspicion of malignancy and guide appropriate referral to specialized neuro-oncology centers. Second, elevated inflammatory markers may prompt clinicians to prioritize surgical scheduling for patients with suspected high-grade lesions, potentially reducing delays in initiating definitive treatment. Third, these biomarkers could inform preoperative counseling by providing patients and families with additional information regarding the probable nature of their lesion, thereby facilitating more realistic expectations and informed decision-making. Fourth, in cases where radiological findings are equivocal, markedly elevated DNI or NLR values might support the decision to proceed with surgical intervention rather than continued observation. Finally, integration of these parameters into preoperative assessment protocols could assist in resource allocation, including the preemptive arrangement of postoperative intensive care, neuro-oncology consultation, and adjuvant treatment planning. It should be emphasized, however, that these biomarkers are intended to complement, rather than replace, conventional diagnostic modalities, and histopathological confirmation remains the definitive standard for diagnosis.

This study has certain limitations. The single-center design and retrospective methodology may restrict the generalizability of the results to patient populations with different demographic and clinical characteristics. Hematological inflammation markers can be influenced by several factors including prior infection, subclinical inflammation, stress response, and medication use. Although corticosteroid and NSAID use did not differ significantly among groups and were included as covariates in regression analyses, the potential subclinical effects of these medications on inflammatory markers cannot be entirely excluded in a retrospective design. Although strict exclusion criteria were applied, it was not possible to fully control for all potential confounding variables. Due to the insufficient number of rare tumor subtypes, subgroup analyses based on histological classification could not be performed. Importantly, tumor volumetric data, the degree of peritumoral edema, and detailed symptom severity scores were not systematically recorded, precluding analysis of the relationship between tumor burden and inflammatory marker elevation. This limitation is particularly relevant for interpreting findings in the benign tumor group, as the magnitude of inflammatory response likely varies with tumor size, edema extent, and associated mass effect. The fact that our cohort comprised exclusively surgical candidates—predominantly symptomatic patients with lesions deemed appropriate for operative intervention—introduces selection bias that may limit generalizability to the broader population of patients with small, incidentally discovered, or conservatively managed benign brain tumors, in whom systemic inflammatory responses would be expected to be minimal or absent. Prognosis and survival outcomes were not assessed, as the study focused solely on diagnostic discrimination. Furthermore, the study did not prospectively evaluate whether implementation of these biomarkers in clinical practice would lead to measurable improvements in patient outcomes, treatment timing, or healthcare resource utilization. Future studies incorporating clinical decision-making endpoints would be valuable to establish the real-world impact of integrating these markers into neuro-oncology workflows. Considering these limitations, validation of the findings through multicenter, prospective studies with larger sample sizes is needed to support the translation of these results into clinical practice.

## 5. Conclusions

In conclusion, this study demonstrates that DNI and NLR are clinically meaningful biomarkers for predicting the likelihood of malignancy in primary brain tumors. The combination of these two parameters further enhanced diagnostic accuracy and highlighted their potential value as noninvasive, low-cost assessment tools during the preoperative period. However, consideration should be given to the influence of age on inflammatory profiles, as it may act as a confounding factor in the interpretation of these markers. Our findings suggest that hematological inflammation parameters may serve as supportive diagnostic tools in neuro-oncology practice, particularly in patients with symptomatic lesions warranting surgical evaluation, while definitive diagnosis must always rely on pathological confirmation. These markers may be particularly useful in guiding surgical prioritization, patient counseling, and referral decisions in settings with limited access to advanced neuroimaging. Prospective studies evaluating the impact of incorporating DNI and NLR into clinical decision-making pathways on treatment timing and patient outcomes are warranted to establish their role in routine neuro-oncology practice.

## Figures and Tables

**Table 1:** Summary of age, sex, laboratory measurements and markers with regard to groups.

	Groups			
	Malign (*n* = 60)	Benign (*n* = 50)	Control (*n* = 30)	*p*
Age, years	57 (51–64)	49 (44–57) *	55.5 (49–64)	**<0.001 ^‡^**
Sex				
Male	26 (43.33%)	20 (40.00%)	16 (53.33%)	0.499 ^§^
Female	34 (56.67%)	30 (60.00%)	14 (46.67%)	
Pathology				
Anaplastic astrocytoma	18 (30.00%)	0 (0.00%) *	-	**<0.001 ^¶^**
Glioblastoma	42 (70.00%)	0 (0.00%) *	-	
Meningioma	0 (0.00%)	32 (64.00%) *	-	
Pituitary adenoma	0 (0.00%)	6 (12.00%) *	-	
Schwannoma	0 (0.00%)	12 (24.00%) *	-	
Symptom	54 (90.00%)	41 (82.00%)	28 (93.33%)	0.258 ^§^
Cognitive/behavioral change	20 (33.33%)	0 (0.00%) *	16 (53.33%) ^#^	**<0.001 ^§^**
Focal deficit	20 (33.33%)	17 (34.00%)	17 (56.67%)	0.071 ^§^
Headache	34 (56.67%)	21 (42.00%)	12 (40.00%)	0.192 ^§^
Nausea/vomiting	23 (38.33%)	0 (0.00%) *	9 (30.00%) ^#^	**<0.001 ^§^**
Seizure	21 (35.00%)	15 (30.00%)	8 (26.67%)	0.698 ^§^
Other	0 (0.00%)	12 (24.00%) *	0 (0.00%) ^#^	**<0.001 ^¶^**
Steroid use	16 (26.67%)	9 (18.00%)	11 (36.67%)	0.176 ^§^
Recent antibiotic use	0 (0.00%)	0 (0.00%)	0 (0.00%)	-
NSAID use	13 (21.67%)	10 (20.00%)	9 (30.00%)	0.563 ^§^
WBC (10^9^/L)	9.76 ± 2.07	7.67 ± 1.73 *	6.34 ± 1.59 *^#^	**<0.001 ^†^**
Neutrophil (10^9^/L)	6.79 (5.81–7.72)	4.92 (3.90–5.89) *	3.73 (3.13–4.42) *^#^	**<0.001 ^‡^**
Lymphocyte (10^9^/L)	1.27 ± 0.50	1.71 ± 0.49 *	1.97 ± 0.61 *	**<0.001 ^†^**
Monocyte (10^9^/L)	0.51 ± 0.18	0.51 ± 0.19	0.50 ± 0.19	0.947 ^†^
Platelet (10^9^/L)	237.65 ± 62.35	238.54 ± 61.16	241.40 ± 63.60	0.964 ^†^
DNI (%)	5.00 ± 1.99	2.51 ± 1.28 *	1.08 ± 0.53 *^#^	**<0.001 ^†^**
Hemoglobin (g/dL)	13.26 ± 1.71	13.02 ± 1.49	13.66 ± 1.76	0.244 ^†^
Hematocrit (%)	40.7 (36.2–42.1)	39.75 (35.2–42.4)	40.65 (35.7–43.8)	0.604 ^‡^
NLR	5.71 (3.99–7.50)	2.63 (2.00–3.51) *	1.76 (1.36–2.56) *	**<0.001 ^‡^**
PLR	181.37 (138.90–259.15)	144.16 (112.16–186.73) *	122.05 (94.07–172.33) *	**<0.001 ^‡^**
MLR	0.45 (0.27–0.57)	0.29 (0.23–0.40) *	0.25 (0.18–0.37) *	**<0.001 ^‡^**
SII (10^3^)	1262.80 (849.48–1873.57)	604.09 (440.49–834.89) *	459.43 (284.00–694.67) *	**<0.001 ^‡^**
CRP (mg/L)	10.13 (7.32–13.38)	4.56 (2.99–5.60) *	8.60 (3.53–10.39) ^#^	**<0.001 ^‡^**
Albumin (g/dL)	3.72 ± 0.33	3.99 ± 0.32 *	3.64 ± 0.30 ^#^	**<0.001 ^†^**

Descriptive statistics are presented using mean ± standard deviation for normally distributed continuous variables, median (25th percentile–75th percentile) for non-normally distributed continuous variables and frequency (percentage) for categorical variables. ^†^ One-way analysis of variance (ANOVA), ^‡^ Kruskal–Wallis test, ^§^ Chi-square test, ^¶^ Fisher–Freeman–Halton test, * significantly different from “Malign” group, ^#^ significantly different from “Benign” group. Statistically significant *p* values are shown in bold. WBC: white blood cells, DNI: delta neutrophil index, NLR: neutrophil-to-lymphocyte ratio, PLR: platelet-to-lymphocyte ratio, MLR: monocyte-to-lymphocyte ratio, SII: systemic immune-inflammation index, CRP: C-reactive protein.

**Table 2:** Performance of markers to discriminate malign and benign tumors; ROC curve analysis.

	Cut-Off	Sensitivity	Specificity	Accuracy	PPV	NPV	AUC (95% CI)	*p*
DNI (%)	>3.50	71.67%	88.00%	79.09%	87.76%	72.13%	0.847 (0.775–0.919)	**<0.001**
NLR	>3.95	76.67%	86.00%	80.91%	86.79%	75.44%	0.850 (0.776–0.923)	**<0.001**
PLR	>161.5	66.67%	66.00%	66.36%	70.18%	62.26%	0.688 (0.589–0.786)	**0.001**
MLR	>0.37	61.67%	74.00%	67.27%	74.00%	61.67%	0.674 (0.573–0.775)	**0.002**
SII (10^3^)	>835.0	78.33%	76.00%	77.27%	79.66%	74.51%	0.829 (0.752–0.905)	**<0.001**
DNI and NLR combination ^a^	-	85.00%	86.00%	85.45%	87.93%	82.69%	0.937 (0.893–0.981)	**<0.001**

ROC: Receiver operating characteristic, PPV: positive predictive value, NPV: negative predictive value, AUC: area under ROC curve, CI: confidence interval, DNI: delta neutrophil index, NLR: neutrophil-to-lymphocyte ratio, PLR: platelet-to-lymphocyte ratio, MLR: monocyte-to-lymphocyte ratio, SII: systemic immune-inflammation index. ^a^ DNI and NLR combination was constructed using logistic regression model. Bold values indicate statistically significant results

**Table 3:** Performance of markers to discriminate malign and benign tumors; age-adjusted ROC curve analysis.

	Sensitivity	Specificity	Accuracy	PPV	NPV	AUC (95% CI)	*p*
DNI (%)	78.33%	76.00%	77.27%	79.66%	74.51%	0.870 (0.804–0.935)	**<0.001**
NLR	75.00%	82.00%	78.18%	83.33%	73.21%	0.870 (0.805–0.935)	**<0.001**
PLR	70.00%	60.00%	65.45%	67.74%	62.50%	0.757 (0.669–0.845)	**<0.001**
MLR	73.33%	68.00%	70.91%	73.33%	68.00%	0.772 (0.686–0.858)	**<0.001**
SII (10^3^)	73.33%	76.00%	74.55%	78.57%	70.37%	0.842 (0.771–0.914)	**<0.001**
DNI and NLR combination ^a^	88.33%	86.00%	87.27%	88.33%	86.00%	0.943 (0.900–0.986)	**<0.001**

ROC: Receiver operating characteristic, PPV: positive predictive value, NPV: negative predictive value, AUC: area under ROC curve, CI: confidence interval, DNI: delta neutrophil index, NLR: neutrophil-to-lymphocyte ratio, PLR: platelet-to-lymphocyte ratio, MLR: monocyte-to-lymphocyte ratio, SII: systemic immune-inflammation index. ^a^ DNI and NLR combination was constructed using the logistic regression model.

**Table 4:** Odds ratios of discriminate malign and benign tumors; logistic regression analysis results.

	Univariable		Multivariable ^a^		
	OR (95% CI)	*p*	OR (95% CI)	*p*	VIF
Age	1.086 (1.038–1.136)	**<0.001**	1.089 (0.995–1.192)	0.063	1.113
Sex, Female	0.872 (0.407–1.868)	0.724			
Symptom, Yes	1.976 (0.651–5.994)	0.229			
Steroid use, Yes	1.657 (0.660–4.160)	0.283			
NSAID use, Yes	1.106 (0.438–2.793)	0.831			
WBC (10^9^/L)	1.822 (1.403–2.366)	**<0.001**	1.878 (1.160–3.041)	**0.010**	1.174
Neutrophil (10^9^/L)	2.178 (1.584–2.995)	**<0.001**		0.108	
Lymphocyte (10^9^/L)	0.167 (0.068–0.408)	**<0.001**		0.693	
Monocyte (10^9^/L)	1.197 (0.155–9.266)	0.863			
Platelet (10^9^/L)	1.000 (0.994–1.006)	0.940			
DNI (%), >3.50	18.549 (6.681–51.502)	**<0.001**	20.667 (3.346–127.642)	**0.001**	1.332
Hemoglobin (g/dL)	1.098 (0.868–1.390)	0.435			
Hematocrit (%)	1.052 (0.967–1.145)	0.235			
NLR, >3.95	20.184 (7.440–54.756)	**<0.001**	21.165 (3.282–136.501)	**0.001**	1.371
PLR, >161.5	3.882 (1.755–8.589)	**0.001**		0.662	
MLR, >0.37	4.579 (2.019–10.382)	**<0.001**		0.369	
SII (10^3^), >835.0	11.449 (4.685–27.978)	**<0.001**		0.978	
CRP (mg/L)	1.400 (1.228–1.597)	**<0.001**	1.522 (1.200–1.931)	**0.001**	1.245
Albumin (g/dL)	0.072 (0.018–0.281)	**<0.001**		0.701	
Nagelkerke R^2^	-		0.839		

^a^ Multivariable analysis was performed via the forward conditional selection method. OR: Odds ratio, CI: confidence interval, VIF: variance inflation factor, WBC: white blood cells, DNI: delta neutrophil index, NLR: neutrophil-to-lymphocyte ratio, PLR: platelet-to-lymphocyte ratio, MLR: monocyte-to-lymphocyte ratio, SII: systemic immune-inflammation index. CRP: C-reactive protein

## Data Availability

The data presented in this study are available on request from the corresponding author due to privacy protections associated with clinical records.

## References

[1] Kaifi R. (2023). A Review of Recent Advances in Brain Tumor Diagnosis Based on AI-Based Classification. Diagnostics.

[2] Grant R., Dowswell T., Tomlinson E., Brennan P.M., Walter F.M., Ben-Shlomo Y., Hunt D.W., Bulbeck H., Kernohan A., Robinson T. (2020). Interventions to reduce the time to diagnosis of brain tumours. Cochrane Database Syst. Rev..

[3] Gilard V., Tebani A., Dabaj I., Laquerrière A., Fontanilles M., Derrey S., Marret S., Bekri S. (2021). Diagnosis and Management of Glioblastoma: A Comprehensive Perspective. J. Pers. Med..

[4] Perkins A., Liu G. (2016). Primary Brain Tumors in Adults: Diagnosis and Treatment. Am. Fam. Physician.

[5] Zhong J.H., Huang D.H., Chen Z.Y. (2017). Prognostic role of systemic immune-inflammation index in solid tumors: A systematic review and meta-analysis. Oncotarget.

[6] Singh N., Baby D., Rajguru J.P., Patil P.B., Thakkannavar S.S., Pujari V.B. (2019). Inflammation and cancer. Ann. Afr. Med..

[7] Yang C., Hu B.W., Tang F., Zhang Q., Quan W., Wang J., Wang Z.-F., Li Y.-R., Li Z.-Q. (2022). Prognostic Value of Systemic Immune-Inflammation Index (SII) in Patients with Glioblastoma: A Comprehensive Study Based on Meta-Analysis and Retrospective Single-Center Analysis. J. Clin. Med..

[8] Zhang C., Wang X., Tian Y., Niu H., Zhang P., Geng J. (2025). Inflammatory markers including NLR, AFR and SII as prognostic factors in neuroblastoma. Sci. Rep..

[9] Choi J.H., Bang C.S., Lee J.J., Baik G.H. (2019). Delta neutrophil index as a predictor of disease severity, surgical outcomes, and mortality rates in gastrointestinal diseases: Rationale for a meta-analysis of diagnostic test accuracy. Medicine.

[10] Moon S., Park Y., Hong C.W., Park S., Sim Y., Ko Y., Park S. (2025). A Usefulness of Delta Neutrophil Index (DNI) for Prediction of 28 Day Mortality in Patients with Pneumonia-Induced Sepsis in the Intensive Care Unit. J. Clin. Med..

[11] Jung W.J., Kim H.S., Cha K.C., Roh Y.I., An G.J., Cha Y.S., Kim H., Lee K.H., Hwang S.O., Kim O.H. (2025). Early Evaluation of Myeloperoxidase and Delta Neutrophil Indices Is Similar to 48 h Sequential Organ Failure Assessment Score for Predicting Multiple Organ Failure After Trauma. J. Clin. Med..

[12] Yune H.Y., Chung S.P., Park Y.S., Chung H.S., Lee H.S., Lee J.W., You J.S., Park I., Lee H.S. (2015). Delta neutrophil index as a promising prognostic marker in out of hospital cardiac arrest. PLoS ONE.

[13] Islam M.M., Satici M.O., Eroglu S.E. (2024). Unraveling the clinical significance and prognostic value of the neutrophil-to-lymphocyte ratio, platelet-to-lymphocyte ratio, systemic immune-inflammation index, systemic inflammation response index, and delta neutrophil index: An extensive literature review. Turk. J. Emerg. Med..

[14] Bozan M.B., Yazar F.M., Kale İ.T., Yüzbaşıoğlu M.F., Boran Ö.F., Azak Bozan A. (2021). Delta Neutrophil Index and Neutrophil-to-Lymphocyte Ratio in the Differentiation of Thyroid Malignancy and Nodular Goiter. World J. Surg..

[15] Bozan M.B., Yazar F.M., Kale I.T., Topuz S., Bozan A.A., Boran O.F. (2022). Immature Granulocyte Count and Delta Neutrophil Index as New Predictive Factors for Axillary Metastasis of Breast Cancer. J. Coll. Physicians Surg. Pak..

[16] Barut O., Demirkol M.K., Kucukdurmaz F., Sahinkanat T., Resim S. (2021). Pre-treatment Delta Neutrophil Index as a Predictive Factor in Renal Cell Carcinoma. J. Coll. Physicians Surg. Pak..

[17] Dharmajaya R., Sari D.K. (2021). Role and value of inflammatory markers in brain tumors: A case controlled study. Ann. Med. Surg..

[18] Manake R., Phillips V., Gangi A., Ravikumar J. (2024). Trends in the Incidence of Brain Cancer: An Observational Study. Cureus.

[19] White M.C., Holman D.M., Boehm J.E., Peipins L.A., Grossman M., Henley S.J. (2014). Age and cancer risk: A potentially modifiable relationship. Am. J. Prev. Med..

[20] Laconi E., Marongiu F., DeGregori J. (2020). Cancer as a disease of old age: Changing mutational and microenvironmental landscapes. Br. J. Cancer.

[21] Li B., Gao B., Zhu H.J., Luwor R.B., Lu J., Zhang L., Kong B. (2024). The Prognostic Value of Preoperative Inflammatory Markers for Pathological Grading of Glioma Patients. Technol. Cancer Res. Treat..

[22] Ahn C., Kim W., Lim T.H., Cho Y., Choi K.S., Jang B.H. (2018). The delta neutrophil index (DNI) as a prognostic marker for mortality in adults with sepsis: A systematic review and meta-analysis. Sci. Rep..

[23] Huang X., Hussain B., Chang J. (2021). Peripheral inflammation and blood-brain barrier disruption: Effects and mechanisms. CNS Neurosci. Ther..

[24] Park K.J., Kang S.H., Chae Y.S., Yu M.O., Cho T.H., Suh J.K., Lee H.K., Chung Y.G. (2010). Influence of interleukin-6 on the development of peritumoral brain edema in meningiomas. J. Neurosurg..

[25] Berhouma M., Jacquesson T., Jouanneau E., Cotton F. (2019). Pathogenesis of peri-tumoral edema in intracranial meningiomas. Neurosurg. Rev..

[26] Park J.H., Byeon H.J., Lee K.H., Lee J.W., Kronbichler A., Eisenhut M., Shin J.I. (2017). Delta neutrophil index (DNI) as a novel diagnostic and prognostic marker of infection: A systematic review and meta-analysis. Inflamm. Res..

[27] Mostofa A.G., Punganuru S.R., Madala H.R., Al-Obaide M., Srivenugopal K.S. (2017). The Process and Regulatory Components of Inflammation in Brain Oncogenesis. Biomolecules.

[28] Wang D.P., Kang K., Lin Q., Hai J. (2020). Prognostic Significance of Preoperative Systemic Cellular Inflammatory Markers in Gliomas: A Systematic Review and Meta-Analysis. Clin. Transl. Sci..

[29] Jarmuzek P., Kozlowska K., Defort P., Kot M., Zembron-Lacny A. (2023). Prognostic Values of Systemic Inflammatory Immunological Markers in Glioblastoma: A Systematic Review and Meta-Analysis. Cancers.

[30] Rusidi H.A., Rosyidi R.M., Wardhana D.P.W., Baskoro W., Ramadhana G.A. (2024). The role of preoperative hematological inflammatory markers as a predictor of meningioma grade: A systematic review and meta-analysis. Surg. Neurol. Int..

[31] Yalon M., Toren A., Jabarin D., Fadida E., Constantini S., Mehrian-Shai R. (2019). Elevated NLR May Be a Feature of Pediatric Brain Cancer Patients. Front. Oncol..

[32] Ashwath K.G., Aggarwal A., Praneeth K., Singla N., Gupta K. (2019). Neutrophil-to-Lymphocyte Ratio: Can It Be Used as an Adjunct Tool to Predict Histopathological Grade of Brain Tumor?. J. Neurosci. Rural. Pr. Pract..

[33] Kayhan A., Korkmaz T.S., Baran O., Kemerdere R., Yeni S.N., Tanriverdi T. (2019). Preoperative Systemic Inflammatory Markers in Different Brain Pathologies: An Analysis of 140 Patients. Turk. Neurosurg..

[34] Brenner A., Friger M., Geffen D.B., Kaisman-Elbaz T., Lavrenkov K. (2019). The Prognostic Value of the Pretreatment Neutrophil/Lymphocyte Ratio in Patients with Glioblastoma Multiforme Brain Tumors: A Retrospective Cohort Study of Patients Treated with Combined Modality Surgery, Radiation Therapy, and Temozolomide Chemotherapy. Oncology.

[35] Zhou J., Tan B., Gao F. (2024). Prognostic values of combined ratios of white blood cells in glioma: A systematic review and meta-analysis. Neurosurg. Rev..

[36] Stepanenko A.A., Sosnovtseva A.O., Valikhov M.P., Chernysheva A.A., Abramova O.V., Pavlov K.A., Chekhonin V.P. (2024). Systemic and local immunosuppression in glioblastoma and its prognostic significance. Front. Immunol..

[37] Xu Y., Li Y., Chen J., Wang L., Wang Z. (2024). Prognostic value of C-reactive protein in patients with glioma: A meta-analysis. Biomark Med.

